# Vaccination hesitancy in the antenatal period: a cross-sectional survey

**DOI:** 10.1186/s12889-018-5389-6

**Published:** 2018-05-02

**Authors:** Paul Corben, Julie Leask

**Affiliations:** 1 Director, North Coast Public Health, Mid North Coast Local Health District, PO Box 126, Port Macquarie, NSW 2444 Australia; 2 Associate Professor, Sydney Nursing School and, Principal Research Fellow, School of Public Health, University of Sydney, 88 Mallett Street (MO2), Sydney, NSW 2050 Australia

**Keywords:** Vaccine hesitancy, Decisional conflict, Antenatal, Decision-making, Immunisation, Midwife

## Abstract

**Background:**

Recent reports of childhood vaccination coverage in Australia have shown steadily improving vaccination coverage and narrowing differences between highest and lowest coverage regions, yet the NSW North Coast consistently has the lowest coverage rates nationally. Better understanding of parents’ vaccination attitudes and actions within this region may guide strategies to improve uptake. The antenatal period is when many parents explore and consolidate vaccination attitudes and so is pivotal for study.

**Methods:**

Women attending public antenatal clinics at six NSW North Coast hospitals completed a 10-min cross-sectional survey capturing stage of decision-making and decisional-conflict as well as vaccination hesitancy, attitudes, intentions and actions. Unscored responses were analysed for individual items. Decisional conflict subscales were scored using published algorithms. For consented children, immunisation status was assessed at 8 months using the Australian Immunisation Register.

For Likert scale items, odds ratios and Fisher’s exact, chi-squared and Chasson’s tests assessed differences between subgroups. Wilcoxon rank-sum tests assessed differences between subgroups for items on scales of 0-to-10 and decisional conflict sub-scale scores.

**Results:**

First-time mothers were 3 times more likely than others (OR = 3.40, 95% CI 1.34–8.60) to identify as unsure, somewhat or very hesitant.

Most respondents (92.2%) wanted their new baby to receive all recommended vaccinations. Many had high or moderate levels of concern about vaccine side effects (25.4%), safety (23.6%) and effectiveness (23.1%).

Increased hesitancy was associated with decreased confidence in the schedule (*p* < 0.001), decreased trust in child’s doctor (*p* < 0.0001), decreased perceived protection from disease (*p* < 0.05) and increased decisional conflict on all measured subscales (*p* < 0.0001). First-time mothers had higher decisional conflict on values clarity, support and uncertainty sub-scales.

By 8 months of age, 83.2% of infants were fully vaccinated. Those with none or a few minor concerns were over 8 times more likely than others to vaccinate on schedule (OR = 8.7, 1.3–56.7).

**Conclusions:**

Importantly this study provides further strong justification to talk with women about vaccination during pregnancy and particularly to ensure that first-time mothers are offered assistance in making these important decisions, where indicated. Further research should focus on optimising the timing, content and delivery style of perinatal interventions.

**Electronic supplementary material:**

The online version of this article (10.1186/s12889-018-5389-6) contains supplementary material, which is available to authorized users.

## Background

Despite the wide acceptance of vaccination and the extensive body of supporting evidence, not all parents choose to vaccinate their children according to recommended schedules [1, 2]. As Kennedy et al. observed “High immunization rates are not the same thing as high confidence in vaccines” [3] and there is growing concern within public health agencies and some sectors of the community that vaccines are losing public confidence [4].

In 2015, the National Vaccine Advisory Committee concluded that as many as one in five United States parents were not fully confident in the safety or importance of vaccines [5]. In the United Kingdom in 2015, 24.5% of surveyed parents were hesitant about vaccines and, for 79% of hesitant parents, confidence issues were the main driver of hesitancy [6]. In Australia, the proportion of parents expressing strong support for immunisation significantly decreased from 86.1% in 2001 to 64.8% in 2009/10 [7, 8]. Should this loss of strong confidence in vaccination translate to behaviour, it could threaten achievement of Australia’s aspirational target of having 95% of children fully vaccinated [9].

Vaccine safety scares, whether factual or fabricated, can erode confidence and motivate rapid and sustained reductions in coverage. Most infamously, the reporting of an alleged link between the measles-mumps-rubella (MMR) vaccine and autism in 1998 resulted in a dramatic fall in vaccine uptake and triggering of outbreaks in the United Kingdom, with MMR coverage falling from 91.8% in 1995–96 to 79.9% in 2003–04, before climbing back to pre-scare levels of 92.1% in 2012–13 [10]. In Australia, reports of febrile reactions including convulsions in children under 5 years of age following vaccination with one of the three available influenza vaccines resulted in a “lasting sense of uncertainty and confusion” amongst some parents [11]. In the era of late modernity where, rather than relying on the advice of experts or authorities, individuals feel obliged to assess risks and modify their behaviours as new information becomes available, episodic undermining of confidence in vaccines could easily threaten high coverage levels [12].

Despite this somewhat bleak outlook, a recent report of childhood vaccination coverage in Australia showed steadily improving rates of vaccination coverage and narrowing of the difference between regions with the highest and lowest coverage [9]. While there is noted variability in the prevalence of recorded conscientious objections to vaccination coverage considered to be associated with clustering of people pursuing “alternate” lifestyles or having shared concerns about allopathic medicine [13, 14], other studies have concluded that likely contributors to variability in coverage include socio-economic status, barriers to access, incomplete recording of vaccinations and missed vaccination opportunities [15–17]. Two recent reports ranked the New South Wales (NSW) north coast lowest amongst Australia’s thirty one Primary Health Network areas across three milestone age groups with 89.8%, 87.2% and 90.3% of children fully vaccinated at one, two and five years of age respectively in 2015/16 [18]. Better understanding of the vaccination attitudes, intentions and actions of parents within this region may help guide strategies to improve uptake of childhood vaccination.

While many studies have sought to unravel the impediments to vaccination decision-making, few have gathered information about when parents make vaccination decisions. Wroe et al. [19] sought the vaccination intentions of 195 women during third trimester of pregnancy and tracked their vaccination decisions. The vast majority (88%) of women in that cohort made their decision during pregnancy and there was a strong association (η = 0.87) between antenatal intentions and vaccination action. Other studies have found that around 28% of parents were undecided about vaccination before the birth of their child and a similar proportion remained hesitant or had doubts about vaccination after the birth of their child, frequently leading to delay or refusal of vaccination [20, 21]. Henrikson et al. observed significant reductions in maternal vaccine hesitancy from birth to 2 years and postulated that, because hesitancy fell as mothers’ confidence in the safety and effectiveness of vaccines grew, the antenatal period and soon after delivery may be ideal times to provide support for parents making vaccination decisions [22]. These studies have provided empirical evidence that the antenatal period is a time when many parents explore and consolidate their vaccination attitudes and therefore presents as a pivotal period for study to improve our understanding of vaccination decisions.

This article reports the attitudes, intentions and vaccination behaviours of pregnant women attending antenatal clinics at public hospitals within the NSW north coast between September 2015 and July 2016.

## Methods

The antenatal vaccination attitudes, intentions and actions reported here were captured in a screening survey intended to identify prospective interviewees who were undecided or hesitant about vaccination of their expected child (see Additional file 1). The survey was part of a mixed methods study with the primary aim of gaining an improved understanding of parents’ experiences of vaccination decision-making during pregnancy and in the first 6 months of life.

Information about the study and the screening survey were distributed by administrative staff and/or midwives to parents attending public antenatal clinics in six hospitals on the NSW north coast. Clinics chose their preferred method to distribute study material. Parents either completed the survey and placed it in a secure box at the clinic or posted it to the research team in a sealed postage-paid envelope. Return of the completed survey was accepted as consent. Additionally, the survey form sought written consent for follow-up of vaccination records and willingness to participate in antenatal and postnatal interviews.

The survey included items from validated instruments measuring stage of decision-making [23], decisional-conflict [24], vaccine hesitancy [21, 25, 26], vaccine beliefs and attitudes [7, 27–29], novel items [30] and basic demographics. O’Connor describes decisional conflict as “a state of uncertainty about a course of action” which may be characterised by “verbalized uncertainty about choices, verbalization of the undesired consequences of alternatives; vacillation between choices, and delayed decision making.” [31]. Vaccine hesitancy may be described as “a delay in acceptance or refusal of vaccination despite availability of vaccination services.” [32].

Where necessary, items were modified to align with Australian parlance eg the word “shot” was replaced with “vaccine” in items drawn from the Parents Attitudes to Childhood Vaccination (PACV) [21]. Items relating to the informed, values clarity, support and uncertainty sub-scales of the Decisional Conflict Scale [31] were included. The survey contained 42 questions, with 36 requiring response on 5-item or 6 –item Likert scales as per scales used by item developers. Two questions adapted from the PACV and one from Kennedy et al. [28] sought a response on the scale 0 to 10, anchored at each end by descriptors. The survey took approximately 10 min to complete.

Descriptive analyses of unscored responses were completed for demographic items and items grouped according to their focus e.g., vaccination attitudes and intentions; risk considerations; social influences or decisional aspects. Decisional Conflict sub-scales were scored according to algorithms used in the scale’s development and validation [24].

Immunisation actions of consenting parents were identified by checking the child’s record on the Australian Immunisation Register (AIR). Immunisation completeness was assessed at 8 months of age in accordance with AIR due and overdue rules and scored as percentage of days unvaccinated in a manner similar to that used by Opel et al. Children were grouped according to percentage of days under-vaccinated [21]. The birth dose of hepatitis B vaccine was not included in completeness calculations and no allowance was made for catch-up schedules. Vaccination completeness was assessed for diphtheria, tetanus, pertussis, polio, haemophilus influenza b, hepatitis B, pneumococcal disease and rotavirus [33].

Analyses were conducted using Microsoft Excel and SAS software Version 9.4 [34]. Odds Ratios (OR), Fisher’s exact test or Chi-squared tests were to assess differences in proportions. The Wilcoxon rank-sum test was used to assess differences between subgroup median scores when responses were measured on scales of 0 to 10 and for the decisional conflict sub-scale scores. Because rank-sum statistics are influenced by sample size and hence are not readily compared across studies, mean score and scale values and normal-approximation confidence intervals are reported for relevant items. Percentages exclude missing values (non-respondents).

The study was approved by the North Coast NSW Human Research Ethics Committee (LNR116) on 24 April 2015.

## Results

Overall findings from the survey about vaccination attitudes, intentions, social influences and risk considerations are summarised in Table 2.

### Demographics

Surveys were completed by 231 expectant mothers attending antenatal clinics between 28 September 2015 and 27 July 2016 and 100 (43%) respondents consented to follow-up of their child’s vaccinations. The 231 respondents represented approximately 5.9% of the estimated births in the region during in the same period and all participating clinics (range: 9–98 per clinic).

Respondents’ age distribution was similar to that of North Coast mothers delivering in 2015 (*p* = 0.404) [35]. Compared to Australian Bureau of Statistics 2011 Census of Population and Housing estimates, the sample included a higher proportion of women completing Year 12 (73.1% vs 55.7%) and a higher percentage (80.8% vs 51.2%) completing a post-secondary qualification (trade certificate, bachelor’s degree or higher) (Table 1).

**Table 1:** Demographics of respondents

Demographic items	Number (%)
Age group (*N* = 222)	
18–24 years	43 (19.4%)
25–29 years	63 (28.4%)
30–34 years	73 (32.9%)
35–39 years	35 (15.8%)
40–44 years	8 (3.6%)
First baby (*N* = 228)	
Yes	80 (35.1%)
No	148 (64.9%)
Trimester completed survey form (*N* = 223)	
1st Trimester	2 (0.9%)
2nd Trimester	85 (38.1%)
3rd Trimester	136 (61.0%)
Highest year of secondary schooling (N = 223)	
Year 12	163 (73.1%)
Year 11	13 (5.8%)
Year 10	39 (17.5%)
Year 9	6 (2.7%)
Other	2 (0.9%)
Post-secondary qualification (*N* = 219)	
Yes, trade certificate or apprenticeship	51 (23.3%)
Yes, other qualification	126 (57.5%)
No, still studying for 1st qualification	8 (3.7%)
No	34 (15.5%)
Highest post-secondary qualification (*N* = 183)	
Professional fellowship qual	3 (1.6%)
Master’s degree	10 (5.5%)
Bachelor’s degree	58 (31.7%)
Assoc degree or diploma	26 (14.2%)
Cert III or Cert IV	74 (40.4%)
Cert II or Cert I	12 (6.6%)

### Vaccination attitudes, intentions and antenatal actions

Support for childhood vaccination was high, with no significant differences in strong support based on parity (*p* = 0.304), highest school level (*p* = 0.184) or having a post-secondary qualification (*p* = 0.826).

**Fig. 1:**
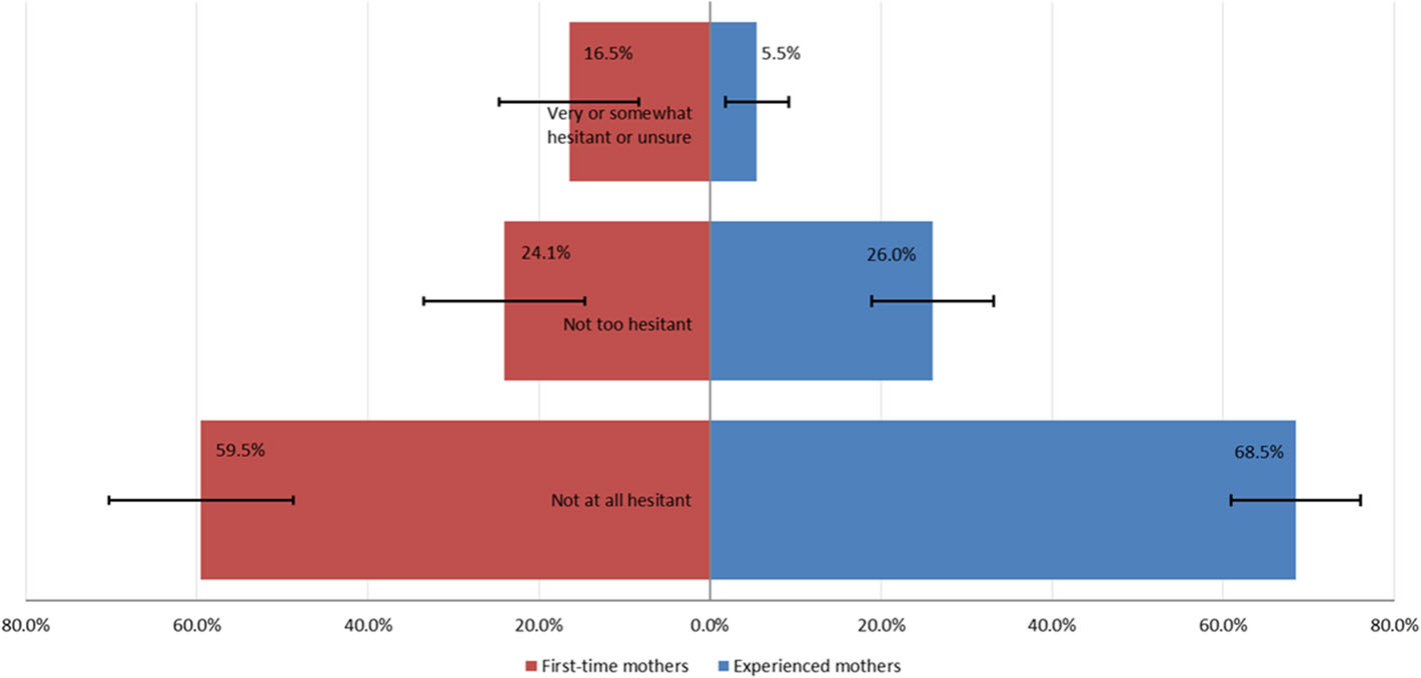
Self-assessed hesitancy about childhood vaccines, expectant mothers NSW north coast 2015–16

Overall, 65.3% of respondents assessed themselves as “not at all hesitant” about childhood vaccination, 25.3% as “not too hesitant”, 3.6% were somewhat hesitant, 2.2% were very hesitant and 3.6% were not sure. First-time mothers were 3 times more likely than experienced mothers (OR = 3.40, 95% CI 1.34–8.60) to describe themselves as unsure, somewhat or very hesitant (Fig. 1 and Table 2).

Strong support for childhood vaccination decreased with increasing self-assessed hesitancy, dropping from 93.2% among ‘not at all hesitant’ respondents to 64.9% of the ‘not too hesitant’ and 28.6% of the very/somewhat hesitant or unsure (Chassan’s test for trend, *p* < 0.005) [36].

Overall 92.2% of respondents indicated that they wanted their new baby to receive all recommended vaccinations, with no significant differences based on parity (*p* = 0.077), level of schooling completed (*p* = 0.242) or completion of a post-secondary qualification (*p* = 0.782). When asked about vaccination intentions and vaccine concerns, 42.5% stated they had no concerns, 50.4% had a few minor concerns, 2.2% had lots of concerns and 4.9% were unsure about vaccinating their baby or would delay or refuse some or all vaccines.

Amongst those who wanted their child to receive all vaccines, those who identified as ‘not too hesitant’ were 8 times more likely (OR = 8.2, 3.7–17.9) to have a few or lots of concerns about vaccine safety than the ‘not at all hesitant’.

While 48.5% of respondents indicated they had received or planned to receive an influenza vaccination during pregnancy, 87.4% indicated they had or planned to receive a pertussis vaccination during pregnancy, with no differences based on parity. Those who reported any level of hesitancy about childhood vaccination were 45% less likely to report having or planning to have influenza vaccine (OR = 0.55, 0.32–0.95).

Those who indicated any level of hesitancy were more likely to report ever delaying a vaccine (OR = 9.2, 1.8–46.6) and more likely to report ever deciding against vaccinating a child (OR = 9.5, 1.0–87.3) for reasons other than illness or allergy.

### Social influences

Social influences were important in respondents’ vaccination decisions. Mothers who expressed any level of hesitancy (compared to ‘not at all hesitant’) were less likely to strongly agree that people important to them supported them to vaccinate their child (OR = 0.21, 0.11–0.41) and less likely to strongly agree that people important to them would vaccinate their own child (OR = 0.21, 0.12–0.37).

However, those who strongly agreed that “access to government family support payments is important in vaccination decisions” were not more likely to be primiparous, (*p* = 0.850), concerned about vaccines (*p* = 0.097) or report any level of hesitancy (*p* = 0.561).

**Fig. 2:**
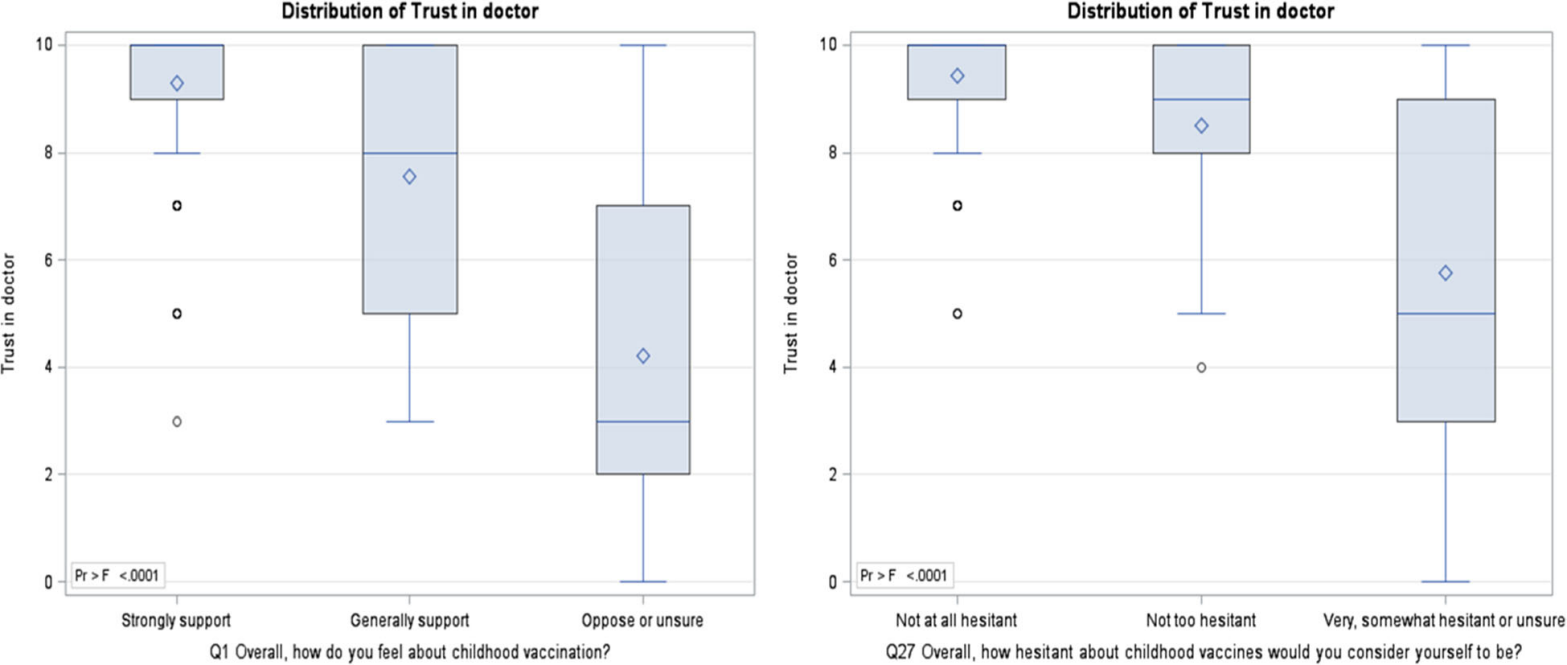
Distribution of Trust in doctor (Q29) by support for vaccination (Q1) and self-assessed hesitancy (Q27)

Overall, respondents indicated a high level of trust in their child’s doctor, with 84.5% rating their trust as 8 or above. There were no differences in trust rating based on parity (*p* = 0.221), level of school completion (*p* = 0.815) or completion of post-secondary qualifications (*p* = 0.225). Respondents who strongly supported childhood vaccination showed higher levels of trust (mean = 9.30, 9.11–9.48) than those who generally supported childhood vaccination (7.56, 6.82–8.29) and those who opposed or were ambivalent (4.22, 1.57–6.88) (*p* < 0.0001). Similarly, those who were ‘not at all hesitant’ reported higher levels of trust (9.43, 9.25–9.61) than the ‘not too hesitant’ (8.51, 8.07–8.95) and the very/somewhat hesitant or unsure (5.76, 4.23–7.30) (*p* < 0.0001) (Fig. 2).

### Risk considerations

Almost all respondents agreed that the benefits of vaccines outweighed the risks, with 69.6% strongly agreeing and a further 21.8% agreeing. Only 2.8% disagreed or strongly disagreed. First-time mothers were less sure about the balance of risks, with 12.0% compared to 2.7% of experienced mothers neither agreeing nor disagreeing that benefits outweigh risks (OR = 4.84, 1.44–16.29) (Table 2). The ‘not too hesitant’ were 88% less likely than the ‘not at all hesitant’ to agree that benefits outweigh risks (OR = 0.12, 0.02–0.61).

**Table 2:** Antenatal vaccination attitudes, intentions, social influences and risk considerations

	Overall % (N)	Primips	Multips	*P* value	Post-secondary qualifs	Other	*P* value	Not at all hesitant	Others	*P* value	Very or somewhat hesitant	Others	*P* value
**Vaccination attitudes, intentions** **& pre-natal actions**													
Qn1 Strongly support childhood vaccination	80.0% (230)	76.0% (79)	81.8% (148)	0.304	81.3% (143)	80.0% (32)	0.826	93.5% (139)	58.1% (74)	< 0.0001	15.4% (13)	83.9% (211)	< 0.0001
Qn26 Want baby to get all recommended vaccines (Yes)	92.2% (204)	86.0% (57)	94.6% (147)	0.077	93.0% (158)	92.1% (38)	0.737	99.2% (133)	73.2% (82)	< 0.0001	33.3% (12)	95.8% (190)	< 0.0001
Qn27 Not at all hesitant	65.3% (225)	59.5% (79)	68.5% (146)	0.189	62.5% (184)	57.8% (45)	0.609						
Qn27 Very/somewhat hesitant or unsure	5.8% (225)	16.5% (79)	5.5% (146)	0.014	12.0% (184)	15.6% (45)	0.616						
Qn 31 Want baby to have all recommended vaccines and have no concerns	42.5% (226)	41.8% (79)	42.9% (147)	0.681	38.7% (186)	43.2% (44)	0.610	60.5% (147)	9.2% (76)	< 0.0001	0.0% (12)	45.5% (211)	0.001
Qn 36 Plan to have flu vaccine (Yes)	48.5% (231)	50.0% (86)	47.4% (152)	0.787	51.4% (185)	36.4% (44)	0.093	55.9% (145)	35.6% (90)	0.003	15.4% (13)	51.7% (209)	0.019
Qn 37 Plan to have pertussis vaccine (Yes)	87.5% (231)	90.7% (86)	88.2% (152)	0.667	90.3% (185)	86.4% (44)	0.421	89.38% (147)	87.5% (88)	0.669	75.0% (12)	90.1% (211)	0.126
Qn24 Ever delayed vaccine for reasons not illness or allergy? (% yes, multips only)	7.5% (147)				7.8% (116)	7.7% (26)	0.588	2.1% (97)	16.3% (43)	0.0008	60.0% (5)	4.5% (134)	0.002
Qn25 Ever decided not to vaccinate child for reasons other than illness or allergy? (% yes, multips only)	3.5% (144)				4.3% (116)	0.0% (24)	> 0.999	1.0% (98)	8.9% (45)	0.0341	66.7% (6)	0.7% (135)	< 0.0001
**Social influences**													
Qn4 Strongly agree “Most people who are important to me think I should get my child vaccinated”	62.7% (220)	57.3% (75)	65.0% (143)	0.304	60.4% (169)	70.0% (40)	0.282	75.9% (141)	39.2% (74)	< 0.0001	35.3% (17)	64.5% (211)	0.034
Qn5 Strongly agree “Most people who are important to me would have their child vaccinated”	63.0% (227)	61.5% (78)	63.7% (146)	0.773	60.7% (173)	71.4% (42)	0.217	75.2% (145)	42.1% (76)	< 0.0001	30.8% (13)	65.9% (208)	0.016
Qn20 Strongly agree “Access to government family support payments is important in my decisions”	16.4% (226)	16.9% (77)	15.8% (146)	0.850	15.0% (173)	22.0% (41)	0.346	14.6% (144)	18.4% (76)	0.561	33.3% (18)	14.9% (215)	0.235
Qn29 All things considered, how much do you trust your child’s doctor? (From 0 = Do not trust at all to 10 = completely trust) Mean (95% CI)	8.8 (8.6–9.1)	8.7 (8.3–9.2)	8.7 (8.4–9.0)	0.990	8.8 (8.5–9.1)	8.5 (8.0–9.2)	0.369	9.4 (9.3–9.6)	7.7 (7.3–8.2)	< 0.0001	5.4 (3.8–6.9)	9.0 (8.8–9.2)	< 0.0001
**Risk considerations**													
Q3 “Because other children are vaccinated it isn’t necessary to have my child vaccinated” –Strongly or moderately agree	9.9% (223)	7.9% (76)	9.0% (145)	> 0.999	9.3% (172)	7.3% (41)	> 0.999	8.3% (144)	9.5% (74)	0.803	16.7% (12)	8.3% (206)	0.281
Q19 “The benefits of vaccines outweigh the risks of vaccines” – Neither agree nor disagree	5.8% (224)	12.0% (75)	2.7% (146)	0.012	7.7% (181)	9.1% (44)	0.759	1.4% (141)	14.3% (77)	0.0003	30.8% (13)	4.4% (205)	0.0041
Q21 “How concerned are you that your child may have a serious side effect from a vaccine?” - Very or somewhat concerned	25.4% (228)	26.6% (79)	24.7 (146)	0.751	25.7% (175)	24.4% (41)	> 0.999	8.3% (145)	55.8% (77)	< 0.0001	76.9% (13)	21.5% (209)	< 0.0001
Q22 “How concerned are you that any one of the childhood vaccines might not be safe?” - Very or somewhat concerned	23.6% (229)	30.4% (79)	19.7% (147)	0.099	244.0% (175)	23.8% (42)	> 0.999	6.8% (146)	54.5% (77)	< 0.0001	84.6% (13)	19.5% (210)	< 0.0001
Q23 “How concerned are you that a vaccine might not prevent the disease” - Very or somewhat concerned	23.1% (226)	21.8% (78)	23.3% (146)	0.868	24.3% (173)	16.7% (42)	0.412	11.7% (145)	43.4% (76)	< 0.0001	61.5% (13)	20.2% (208)	0.002
Q28 How sure are you that following the recommended vaccine schedule is a good idea for your child? (0 = not at all sure, 10 = completely sure) Mean (95% CI)	8.83 (8.54–9.12)	8.49 (7.89–9.09)	9.01 (8.70–9.33)	0.564	8.91 (8.59–9.24)	8.38 (7.58–9.18)	0.176	9.73 (9.62–9.84)	7.17 (6.47–7.86)	< 0.0001	3.77 (2.02–5.52)	9.15 (8.91–9.39)	< 0.0001
Q30 If children in Australia are not vaccinated, how likely do you think they are to get a disease that vaccines prevent? (0 = not at all likely, 10 = very likely) Mean (95% CI)	7.14 (6.81–7.47)	7.17 (6.57–7.79)	7.13 (6.74–7.52)	0.887	7.18 (6.83–7.53)	6.66 (5.68–7.63)	0.240	7.57 (7.19–7.95)	6.38 (5.77–6.99)	0.001	3.83 (2.18–5.49)	7.35 (7.02–7.67)	< 0.0001

*P* value is for Fisher’s exact test for 2 × 2 contingency table. Figures shown are percentage and total number of respondents as % (N)

Primips=first-time mothers; Multips=experienced mothers

About a quarter of respondents had high or moderate levels of concern about potential side effects (25.4%), concerns childhood vaccines might not be safe (23.6%) and concerns that a vaccine might not prevent disease (23.1%), with no differences based on parity or highest year of schooling (Table 2). Table 3 summarises increasing likelihood of concern about vaccine safety, vaccine side-effects and vaccine effectiveness as respondents’ levels of hesitancy increased.

**Table 3:** Odds ratios - vaccine concerns and decisional conflict by self-assessed hesitancy, expectant mothers

	Not at all hesitant (referent)	Not too hesitant^a^	Very or somewhat hesitant or unsure^a^
Vaccine concerns			
Very/somewhat concerned vaccine might have side effect	Referent	11.1 (5.0–24.4)	27.7 (9.1–84.6)
Very/somewhat concerned vaccine might be unsafe	Referent	11.8 (5.1–27.0)	43.5 (13.2–143.3)
Very/somewhat concerned vaccine might not prevent disease	Referent	4.7 (2.2–9.8)	10.0 (3.7–27.3)
Decisional conflict sub-scale ^b^			
Informed subscale (score ≥ 37.5)	Referent	7.9 (3.2–19.3)	12.0 (4.3–33.5)
Values clarity subscale (score ≥ 37.5	Referent	3.7 (1.5–8.8)	11.4 (4.4–29.5)
Support sub-scale (score ≥ 37.5)	Referent	13.1 (2.7–62.9)	43.7 (9.0–213.0)
Uncertainty subscale (score ≥ 37.5)	Referent	17.4 (3.8–80.4)	85.4 (17.7–413.7)

^a^Odds ratio (95% confidence interval) – referent = “Not at all hesitant”

^b^Decisional conflict sub-scale scores ≥37.5 are associated with decision delay or feeling unsure about implementation

Overall, respondents were confident that following the recommended vaccination schedule was good for their child with 85.4% rating 8 or more (on scale 0–10) and averaging 8.83 (8.54–9.13). There was a downward gradient in respondents’ confidence in the schedule with increasing hesitancy (*p* < 0.001) and with decreasing overall support for vaccination (*p* < 0.0001). More hesitant respondents reported lower likelihood of contracting a vaccine-preventable disease in Australia if unvaccinated with significant differences in perceived likelihood of disease between the ‘not at all hesitant’ and ‘not too hesitant’ (*p* = 0.046) and between the ‘not too hesitant’ and ‘very/somewhat hesitant and unsure’ (*p* = 0.019).

### Decision-making aspects

At the time of completing the survey, 80.7% (75.6%–85.8%) of respondents had made up their mind and were unlikely to change and an additional 7.9% (4.4%–11.4%) had made a decision but were willing to reconsider. First-time mothers (77.5%) were less likely than experienced mothers (94.5%) to have made their decision (including those willing to reconsider) at the time of completing the survey (*p* = 0.0003).

About one-third (35.5%) of first-time mothers completing the survey up to the end of second trimester had not made a decision compared to 5.8% of experienced mothers (*p* = 0.0015). For those completing the survey during their third trimester, the proportions of ‘undecided’ mothers decreased to 14.9% for first-timers and to 4.4% for experienced mothers (*p* = 0.0086).

**Fig. 3:**
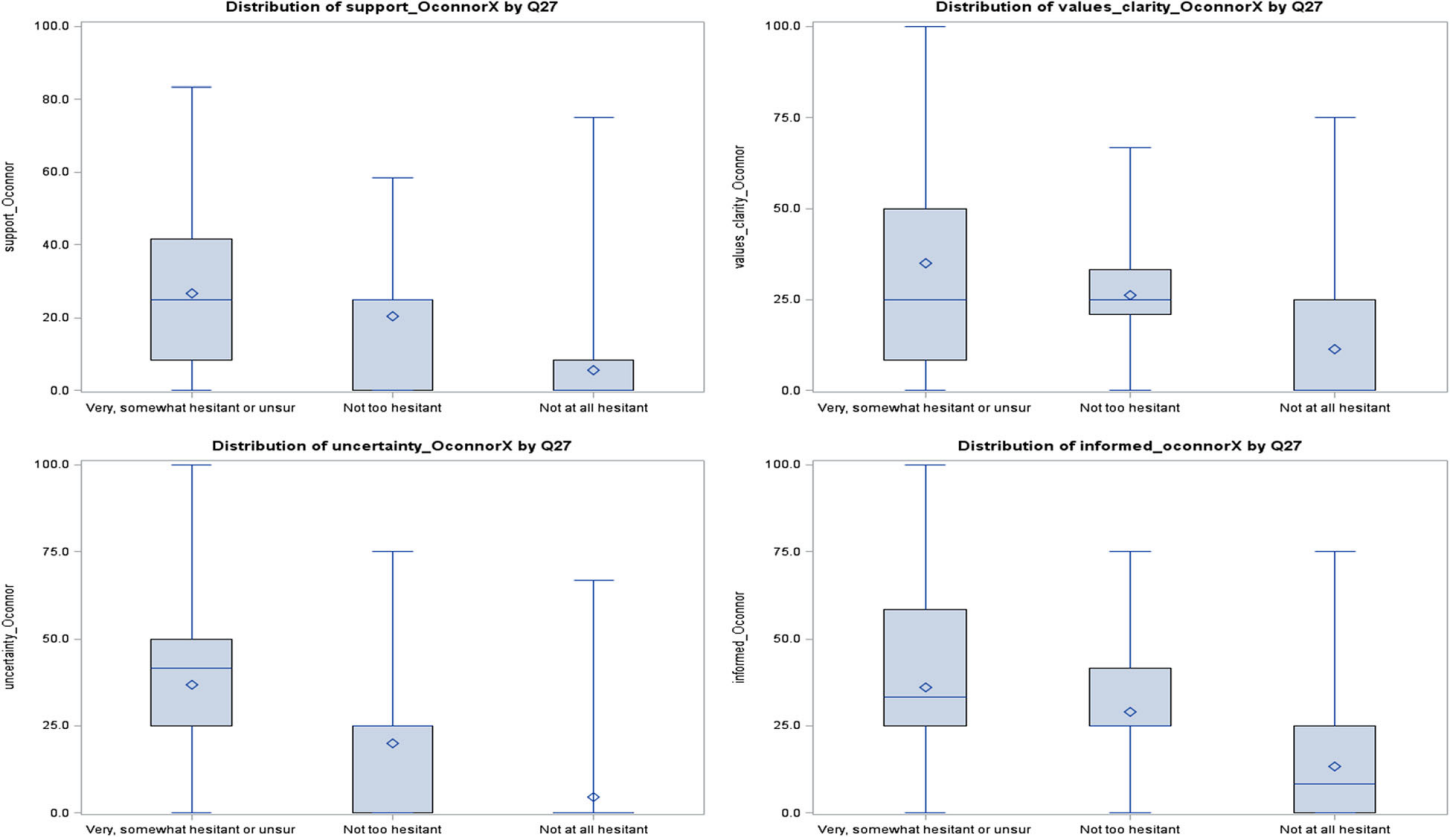
Decisional conflict subscales by self-rated vaccine hesitancy. Note: Mean scores are displayed as these are more readily interpreted and are not affected by sample size as are rank sum statistics. Decisional conflict sub-scale scores ≥37.5 are associated with decision delay or feeling unsure about implementation

Table 3 reflects the strong influence of increasing levels of self-assessed hesitancy on decisional conflict sub-scale scores. The ‘not at all hesitant’ had significantly lower median scores (*p* < 0.0001) on each of the four decisional conflict subscales than those who identified as ‘not too hesitant’ (Fig. 3). The ‘not too hesitant’ had similar scores to those rating themselves as ‘very/somewhat hesitant or unsure’ for the informed (*p* = 0.3856), values clarity (*p* = 0.3513) and support (*p* = 0.3165) sub-scales but lower median scores on the uncertainty subscale (*p* = 0.0169).

While first-time and multiparous mothers had similar median scores on the informed sub-scale (*p* = 0.1306), first-time mothers displayed higher decisional conflict on the other three sub-scales (values clarity *p* = 0.0092, support *p* = 0.0315 and uncertainty *p* = 0.0033).

### Immunisation outcomes

One hundred women (43.7% of respondents) consented to access of their babies’ vaccination records, with AIR records for 101 infants able to be accessed (including two sets of twins). By 8 months of age, 83.2% of the infants were fully vaccinated within 30 days of the recommended date for each vaccine (i.e., zero days under-immunised) and a further 12.1% had immunised their baby after minor delay (< 10% of follow-up days).

There was no difference detected in vaccination timeliness of babies of first-time mothers and experienced mothers (*p* = 0.242) nor between those who considered themselves ‘not at all hesitant’ and others (*p* = 0.705). Those with no concerns or a few minor concerns were over 8 times more likely to vaccinate on schedule than others (OR = 8.7, 1.3–56.7) and those with a few concerns were just as likely to vaccinate on time as those with no concerns (OR = 1.4, 0.5–4.5). Similar to Danchin et al., we detected no consistent correlation between uptake of maternal vaccines and infants’ vaccination timeliness, with no difference in the odds of on-time infant vaccination for those choosing maternal influenza vaccination compared to those without (OR = 1.78,0.63–4.99), on-time infant vaccination and maternal pertussis vaccination (OR = 0.71, 0.08–6.34), or on-time infant vaccination for those choosing both maternal influenza and pertussis vaccination (OR = 1.65, 0.59–4.64) [37].

There were no systematic differences between those consenting to vaccination follow-up and others according to parity, support for vaccination, trust in doctors, clinic group, concerns about vaccine side effects, self-assessed hesitancy, vaccination intention or stage of decision. Lack of variability in the vaccination actions of the respondents may reflect selection bias, with those completing surveys being more supportive of vaccination.

## Discussion

This study confirms that most expectant mothers make decisions about vaccination before or during their pregnancy. As in similar studies [19, 37], we found that the overwhelming majority (81%) of women attending antenatal clinics reported they had made a firm decision about vaccination of their expected child and a further 8% indicated that while they had made a decision, they might reconsider it before acting. Fewer first-time mothers (77.5%) than experienced mothers (94.5%) reported making a decision before or during pregnancy. About a third (35.5%) of first-time mothers were undecided during their second trimester and by third trimester one in seven (14.9%) first-time mothers had still not decided about vaccinating their newborn. First-time mothers were 5 times more likely to be unsure that the benefits of vaccines outweigh their risks and 3 times more likely to declare they were somewhat/very hesitant or unsure about vaccinating their child.

We believe this is the first study that has assessed the decisional conflict experienced by pregnant women about vaccination of their unborn child. First time mothers had significantly higher median scores on three of four decisional conflict sub-scales (values clarity, support and uncertainty sub-scales), placing them at higher risk of decisional delay or feeling unsure about implementing their decisions.

These findings suggest there may be benefit from engaging with women about vaccination decisions during family planning and early in pregnancy and particularly so for women expecting their first child. Midwives are uniquely well-placed to support expectant and new mothers to make informed decisions about maternal and infant vaccination. However, some midwives find aspects of a vaccination advocacy role challenging due to the need to reconcile professional preferences for women’s autonomy in decision-making or, for a significant minority, holding concerns about the safety, necessity and timing of vaccinations but working within the context of strident public health advocacy for timely vaccination of infants [38–40]. Despite these challenges, significant positive associations between provision of immunisation education and support during the antenatal period and uptake of childhood immunisation have been demonstrated in a range of settings including within a cluster randomised trial in Japan and cross-sectional studies in Nigeria and Cameroon [41–43].

While most (80%) of respondents were strong supporters of vaccination and over 90% agreed that the benefits of vaccines outweigh the risks, a quarter expressed concern that a vaccine may not be safe, that their child could suffer serious side-effects or that the vaccine may not protect against the targeted diseases. Compared to the “not at all” hesitant, other pregnant women were 14 times more likely to be concerned about vaccine safety, 16 times more likely to be concerned about side-effects and 6 times more likely to have concerns about vaccine effectiveness. We found that even those who were “not too hesitant” expressed much higher levels of concern about safety, side-effects and effectiveness.

We also found that any level of stated hesitancy was associated with an 80% reduction in the likelihood of having important social contacts who supported vaccination and a 9-fold greater likelihood of ever delaying or refusing a child’s vaccination for reasons other than illness or allergy. These findings portray the complexity and impact of psychosocial and other factors on pregnant women’s immunisation attitudes, intentions and behaviours [44]. The findings also highlight the frailty underlying high uptake of vaccines and high levels of support for vaccination despite underlying safety concerns [3]. This confidence gap poses a risk to high coverage should a vaccine safety scare emerge and reflects the interplay between public confidence in vaccination and vaccine hesitancy, particularly in response to vaccine safety scares [45].

During the development of our survey, the Australian government announced its “No Jab, No Pay” policy that removed non-medical exemptions from eligibility criteria for certain family assistance payments [46]. Consequently, we included a question to gauge the importance of such policies in participants’ vaccination decisions. In our sample, we found no association between importance of such policies with levels of concern about vaccines or self-assessed hesitancy. Despite claims of wide and significant impact of the policy since its introduction, it seems the policy will struggle to achieve its objective of addressing vaccine refusal and that other approaches will be needed to address hesitancy and refusal.

We observed large, statistically significant and consistent differences between those who described themselves as ‘not too hesitant’ and the ‘not at all hesitant’ and frequently we found no significant difference between the very/somewhat hesitant or unsure and the ‘not too hesitant’ group. This is of interest as the PACV scoring algorithm combines responses of ‘not at all hesitant’ and ‘not too hesitant’ as the non-hesitant response and these categories add zero to the overall hesitancy score, suggesting that this may reduce the sensitivity and specificity of the measure.

While there is need for robust and comprehensive measures of vaccine hesitancy for use in research settings, there is an ongoing need for a simple, sensitive and specific tool for routine use by immunisation providers. Our findings suggest that an algorithm combining a question about the stage of decision with another about self-rated hesitancy may provide a simple and pragmatic approach for use in busy service settings. The algorithm could simply identify parents who reflected any stated level of hesitancy or who have not yet made a final decision about vaccinating their newborn child. Such a tool could be evaluated for its capacity to help professionals identify when parents might want further engagement.

The optimal timing of screening for vaccination hesitancy is unknown. One view would suggest screening be conducted as early as possible during family planning and pregnancy, especially for first-time parents, to allow maximum time to resolve parents’ concerns. However, we have identified that higher rates of hesitancy in early pregnancy appear to resolve naturally for most mothers as they near birth. Nevertheless, with 15% of primiparous women remaining hesitant there appears to be potential to target interventions in the antenatal period to improve vaccination timeliness. In particular, in our sample those with more concerns were less likely to be timely with vaccination. Additionally, it is not entirely clear what the interventions should involve. Danchin et al. recommended that additional research was needed to identify items that highlight parents’ vaccine safety concerns as these appear to feed hesitancy and decisional delay [37]. Vannice et al. found that providing information increased positive maternal attitudes and beliefs about vaccine safety and confidence but no change in perceived necessity for vaccination. Participants in that study expressed a clear preference for receipt of information before vaccinations were due, either during pregnancy or soon after delivery [47]. In a cluster-randomised controlled trial, Saito and colleagues found that mothers who received a series of short, interactive information sessions during pregnancy, soon after delivery and when the baby was 1 month old reported improved positive injunctive social and descriptive norms (moral perceptions of what most people do and what an individual should do, respectively) and increased their perceptions of vaccine benefits [48] and were more likely than controls to vaccinate their child with mandated, but not voluntary, vaccines [43]. However, the approach taken and content of information sessions needs to be chosen carefully. ‘Knowledge deficit’ approaches to address vaccine hesitancy have not been successful, [49] and poorly executed attempts to correct misinformation can backfire [50]. Additionally, debate persists about whether presumptive approaches are more successful than participatory ones [51, 52]. Some studies have found that introducing information that challenges existing beliefs can increase deliberation, perhaps delaying decisions indefinitely. Consequently, it is likely that optimal strategies will need to tailor information and approach to individuals’ needs and thinking styles – clearly a significant challenge in busy clinical settings [44, 53]. Regardless, this study’s findings suggest the perinatal period is a potentially fruitful time for working with parents to prevent future vaccine delay and rejection. For this to occur, optimised interventions will need to be acceptable to those delivering care, including midwives, GPs and other providers of antenatal care and education and equip providers with tools to address concerns about vaccination whilst maintaining parents’ trust [54].

This study has some limitations. Our wish to reduce respondent burden and our focus on screening for undecided parents meant that we did not retain all PACV items nor the items for the effective decision sub-scale. Consequently, we were unable to calculate summary hesitancy and decisional conflict measures. Due to the methods used to distribute the survey through the clinics, women with pro-vaccination attitudes may have been more inclined to complete the survey and this may have resulted in an elevated prevalence of pro-vaccination attitudes and limited variability in vaccination uptake, reducing our ability to identify important contributors to vaccination hesitancy amongst pregnant women. The study allowed clinics to choose their own method of survey distribution. We noted very low response rates amongst clinics that used passive means to distribute surveys and collect responses, reducing representativeness from across the region. Those clinics that actively encouraged mothers to complete and return the survey whilst in the waiting room had very much higher return rates.

## Conclusion

We have documented important contributions to a more complete understanding of vaccination decision-making by pregnant women, highlighted gradients of vaccine hesitancy not previously reported and demonstrated associations between decisional-conflict sub-scales and vaccination attitudes, behaviours and actions. Importantly this study provides further strong justification to talk with women about vaccination during pregnancy and particularly to ensure that first-time mothers are offered assistance in making these important decisions. To accommodate the complexity of these settings, further research should focus on optimising the timing, content and delivery style of perinatal interventions that are acceptable to those providing care and that address the specific concerns of parents who are unsure about vaccinating their newborn child.

## Additional file

Additional file 1: Vaccine Decision Journeys, Survey instrument, instructions and consent form. (PDF 587 kb)

## Abbreviations

AIR: Australian Immunisation Register
NSW: New South Wales (a state on the east coast of Australia)
OR: Odds Ratio
PACV: Parents Attitudes to Childhood Vaccination
