# Supplementary material for: Vaccination hesitancy in the antenatal period: a cross-sectional survey
Source: BMC Public Health. 2018 May 2;18:566. doi: 10.1186/s12889-018-5389-6 (PMC5930425; doi:10.1186/s12889-018-5389-6)
Supplement: Supplementary file 1 — Vaccine Decision Journeys, Survey instrument, instructions and consent form. (PDF 587 kb) [file 12889_2018_5389_MOESM1_ESM.pdf]

North Coast Public Health
Covering both Mid North Coast & Northern NSW Local Health Districts

Vaccine decision journeys

Survey Instructions
Please answer as many of the questions in the survey as you can.

Please provide the single response for each question or statement that best reflects your opinion or how you feel today. Some questions ask you to rate how much you agree or disagree on a scale from strongly disagree to strongly agree and others ask you rate your response on a scale of 0 to 10. For each question put an **X** in the box that best describes your response to the statement. There are no right or wrong answers.

If you agree to allow the researchers to check your baby's vaccination status at around 6 months of age please don't forget to provide the contact details of your doctor and/or nurse immuniser **ON THE BACK PAGE** after answering the survey questions

Please answer the questions as they relate to the baby you are pregnant with now

	(*Select one option only*)	Strongly oppose	Generally oppose	Neither support nor oppose	Generally support	Strongly support
Q1	Overall, how do you feel about childhood vaccination?					

Parents who are expecting a baby will make decisions about vaccination.

Q2 At this time, regarding the decision about vaccinating your baby, would you say you … *Mark one row only*	
	haven't begun to think about the choices
	haven't begun to think about the choices, but am interested in doing so
	are considering the options now
	are close to selecting an option
	have already made a decision but am willing to reconsider
	have already made a decision and am unlikely to change my mind

Q2 Stage of decision making©, AM O'Connor 2000

Mark only one box in each row under the response that best reflects how you feel about the statement today

		Strongly Disagree	Moderately Disagree	Slightly Disagree	Slightly Agree	Moderately Agree	Strongly Agree
Q3	Because other children are vaccinated, it isn't necessary to have my child vaccinated						
Q4	Most people who are important to me think I should get my child vaccinated						
Q5	Most people who are important to me would have their child vaccinated						

Please read the following comments some people make when deciding about vaccinating their child. Please show how strongly you agree or disagree with these comments by placing an X in the column that best shows how you feel about deciding about vaccinating your child.

In making the decision about vaccines for my child		Strongly disagree	Disagree	Neither agree nor disagree	Agree	Strongly agree
Q6	I know which options are available to me					
Q7	I know the benefits of each option					
Q8	I know the risks and side effects of each option					
Q9	I am clear about which benefits matter most to me					
Q10	I am clear about which risks and side effects matter most to me					
Q11	I am clear about which is more important to me (the benefits or the risks and side effects)					
Q12	I have enough support from others to make a choice					
Q13	I am choosing without pressure from others					
Q14	I have enough advice to make a choice					
Q15	I am clear about the best choice for me					
Q16	I feel sure about what to choose					
Q17	The decision is easy for me to make					
Q18	I have given a lot of thought to my decision concerning vaccination for my child					
Q19	The benefits of vaccines outweigh the risks of vaccines					
Q20	Access to government family support payments is important in my decisions about fully vaccinating my child					

Q6-Q17 O'Connor AM. *Validation of a decisional conflict scale*. Med Dec Making 1995;15(1):25-30

Mark only one box in each row under the response that best reflects how you feel today

		Very concerned	Somewhat concerned	Not sure	Not too concerned	Not concerned at all
Q21	How concerned are you that your child might have a serious side effect from a vaccine?					
Q22	How concerned are you that any one of the childhood vaccines might not be safe?					
Q23	How concerned are you that a vaccine might not prevent the disease?					

Mark only one box in each row under the response that best reflects your experience or intentions

		Yes	No	Don't know	Not applicable I am having my first baby
Q24	Have you ever delayed having a child of yours get a vaccine for reasons other than illness or allergy?				
Q25	Have you ever decided not to have a child of yours get a vaccine for reasons other than illness or allergy?				
Q26	When you have had your baby, would you want him/her to get all the recommended vaccines?				

Mark only one box in each row under the response that best reflects how you feel today

		Very hesitant	Somewhat hesitant	Not sure	Not too hesitant	Not at all hesitant
Q27	Overall, how hesitant about childhood vaccines would you consider yourself to be?					

For the next 3 questions indicate your answer on the scale from 0 to 10 using the guide provided.

		0	1	2	3	4	5	6	7	8	9	10
Q28	How sure are you that following the recommended vaccine schedule is a good idea for your child? (*From* 0 = not at all sure *to* 10 = completely sure)											
Q29	All things considered, how much do you trust your child's doctor? (*From* 0= Do not trust at all *to* 10= completely trust)											
Q30	If children in Australia are not vaccinated, how likely do you think they are to get a disease that vaccines prevent? (0= not at all likely, 10=very likely)											

Q31 Which of the following statements best applies to you today? *Mark one row only*	
	I will allow my baby to have all of their recommended vaccines and have NO concerns about the safety of those vaccines
	I will allow my baby to have all of their recommended vaccines and have A FEW MINOR concerns about their safety
	I will allow my baby to have all of their recommended vaccines but have A LOT OF concerns about their safety
	I will allow my baby to have some vaccines on time but there are some I prefer to delay or not have at all
	I will not allow my baby to have any vaccines
	I am unsure about vaccinating my baby

Lastly, we'd like to collect some information about you and your baby:

Q32 What is today's date? DD / MM / YYYY

Q33 What date is your baby due to be born? DD / MM / YYYY

Q34 Will this baby be your first child? ☐ Yes ☐ No

Q35 If this is not your first child, how many other babies have you ever given birth to? _______ (number)

Q36 Are you planning to have or already had a vaccination against influenza (the flu) during this pregnancy? ☐ Yes ☐ No

Q37 Are you planning to have or already had a vaccination against whooping cough (pertussis) during this pregnancy? ☐ Yes ☐ No

For the following questions, place an X in the box that best describes you (Mark just one box only please)

		In hospital	At home	Undecided	Other	Prefer not to say
Q38	Where are you planning to give birth?					

		18-24 years	25-29 years	30-34 years	35-39 years	40-44 years	45+ years	Prefer not to say
Q39	What was your age on your last birthday?							

		Year 12 or equivalent	Year 11 or equivalent	Year 10 or equivalent	Year 9 or equivalent	Year 8 or below	Did not go to school	Prefer not to say
Q40	What is the highest year of primary or secondary school you have *completed*?							

		Yes, trade certificate / apprenticeship	Yes, other qualification	No, still studying for first qualification	No
Q41	Have you completed any educational qualification (including a trade certificate)?				
		Please answer question Q42 below		Thank you for completing the survey	

		Doctorate (PhD, EdD, LLD etc)	Professional fellowship qualification	Master degree	Bachelor degree	Associate Degree or diploma	Cert III or Cert IV	Cert II or Cert I	Trade Cert
Q42	If you answered 'Yes' to Q41, what is the level of the highest qualification that you have completed?								

☺ THANK YOU FOR COMPLETING THE SURVEY ☺

If you agree to allow the researchers to check your baby's vaccination status at around 6 months of age please don't forget to provide the contact details of your doctor and/or nurse immuniser

ON THE NEXT PAGE

Consent for follow-up of vaccination status

If you consent to allowing the researchers to check your baby's vaccination records at 6 months of age please provide the following information and sign below:

Name of hospital where baby is planned to be delivered or name of midwife if home delivery planned	
Name of medical practice or community health centre	
My doctor's/immunisation nurse's name	
My doctor's/immunisation nurse's phone number	
My doctor's/immunisation nurse's address	
My home or mobile phone number	

I, ______<first name________________, ____________family name> ___________________________, of,

____________________< home address) ____,

provide consent for Paul Corben of the North Coast Public Health Unit to access:

- my child's birth records held by the hospital and/or midwife named above, <u>and</u>
- my child's vaccination records held by my doctor/nurse named above, <u>and</u>
- my child's vaccination records on the Australian Childhood Immunisation Register.

Signature: ________________________________ Date: _____________________

Witness name: ____________________________ Witness signature: ____________________

Interest in being interviewed about vaccination decisions

If you would like to participate in **<u>the interviews</u>** (one before your baby is born and the second interview when your baby is about 6 months old) please provide your contact details below:

Your home telephone :	
Your mobile telephone:	
Your email address:	
Your home address	

I ………………………………………………………………………………………….. (print name)

agree to be invited to take part in interviews about making decisions about vaccination with my partner or other support person. I understand that **if we are interviewed we will be provided a separate information and consent form prior to the first interview**.
